# 3D Imaging of Striatal Transplants in a Small Animal Model of Huntington’s Disease

**DOI:** 10.3390/neurolint15030057

**Published:** 2023-07-24

**Authors:** Elisabeth Schültke, Bernd R. Pinzer, Marco Stampanoni, Laura Harsan, Mátè Döbrössy

**Affiliations:** 1 Department of Radiooncology, Rostock University Medical Center, 18059 Rostock, Germany; 2 Laboratory for Optical 3D Metrology and Computer Vision, University of Applied Sciences Kempten, 87435 Kempten, Germany; 3 Paul Scherrer Institute, 5232 Villigen, Switzerland; 4 ICube Laboratory (Engineering Science, Computer Science and Imaging Laboratory) and Biophysics and Nuclear Medicine Department, Faculty of Medicine, University of Strasbourg, 67412 Strasbourg, France; harsan@unistra.fr; 5 Department of Stereotactic and Functional Neurosurgery, Freiburg University Medical Center, 79106 Freiburg, Germany; mate.dobrossy@uniklinik-freiburg.de; 6 Faculty of Biology, University of Freiburg, 79106 Freiburg, Germany

**Keywords:** Huntington’s disease/Huntington disease, imaging, phase contrast, neurotransplantation, models, small animal, synchrotrons

## Abstract

High-resolution imaging in small animal models of neurologic disease is a technical challenge. In a pilot project, we have explored a non-destructive synchrotron imaging technique for the 3D visualization of intracerebral tissue transplants in a well-established small animal model of Huntington’s disease. Four adult female Sprague Dawley rats each received injections of 0.12 M quinolinic acid (QA) into two target positions in the left striatum, thus creating unilateral left-sided striatal lesions similar to those frequently seen in patients suffering from Huntington’s disease. One week after lesioning, the animals received transplants prepared from whole ganglionic eminences (wGEs) obtained from 13- to 14-day-old rat embryos. Of the four lesioned animals, three received transplants of GNP-loaded cells and one animal received a transplant of naïve cells, serving as control. Post-mortem synchrotron-based microCT was used to obtain images of the neurotransplants. The images obtained of GNP-loaded tissue transplants at the synchrotron corresponded in size and shape to the histological images of transplants developed from naïve cells. Thus, we conclude that non-destructive synchrotron imaging techniques such as phase-contrast imaging are suitable to obtain high-resolution images of GNP-loaded tissue transplants.

## 1. Introduction

Current therapies for patients with Huntington’s disease (HD), an autosomal dominant neurodegenerative disease that typically becomes symptomatic between the third and fifth decade of life, are symptom-oriented. While such therapies can improve the patients’ quality of life temporarily by alleviating the symptoms of their progressive functional decline, the disease itself will nevertheless progress. Over the last several decades, potentially disease-modifying approaches have been tested at the pre-clinical level in the laboratory, and some even made it into clinical trials [1]. Neurotransplantation aims to achieve functional regeneration through the replacement of lost cells [2,3]. Possible sources for the graft material include fetal neural progenitor cells [4], mesenchymal stem cells (MSC) [5,6] and induced pluripotent stem cells (iPSC) [7,8]. The transplantation of human fetal tissue has shown to delay disease progression and stabilize cognitive function [9], and the surgical procedure is considered safe [9,10]. However, there has been a rare report of a patient where the transplanted material proliferated more extensively than anticipated, resulting in symptomatic tumor formation [11]. Thus, if a method could be developed to track the implanted cells, the early detection of such overgrowth could save the patient from becoming symptomatic, and the tumor could be removed at an early stage. Similarly, the migration of the implanted cells out of the intended target location could be detected. In an analogy of tumor formation at the transplantation site, early surgical removal would be an option. Furthermore, the option to track the migration of small cell clusters would be of scientific interest, informing about otherwise unknown migration patterns of transplanted cells. In order to test this approach, we have modified a small animal model of Huntington’s disease.

Several types of small animal models to simulate Huntington’s disease have been developed, based either on genetical engineering or on striatal lesioning [12,13]. The cells used for the transplantation models are typically sourced from the whole ganglionic eminence of embryonic rodents. The quinolinic acid (QA) lesioning model chosen for our study has been well-characterized for its histological and biochemical similarities with human HD as well as for locomotor deficits [14,15]. It has been shown that functional recovery correlates with the amount of DARPP-32 (dopamine and adenosine 3′,5′-monophosphate-regulated phosphoprotein with a molecular weight of 32 kDa) striatal projection neurons [16]. Although the quantity of DARPP-32-positive striatal-like neurons increases with the number of deposits of transplanted material [17], for this proof-of-principle study, we have chosen to work with only two deposits per animal. 

With a difference of several orders of magnitude between the brain volume in human patients and small animal models, it is challenging to obtain images of neurotransplants in the same detail as in human patients. To appreciate this challenge, one must consider that with a ratio of 1:40, the ratio between the brain weight and body weight is very similar in both adult rodents and humans [18]. However, while the volume of the adult human brain varies between 1000 cm^3^ and 1500 cm^3^ [19], the volume of an adult rat brain may only average 600 mm^3^, equaling 0.6 cm^3^ [20]. Assuming a median human brain volume of 1200 cm^3^, the brain volume of a rat contains only 0.05% of this volume, being smaller than the human brain by more than three orders of magnitude. Subsequently, the number of cells deposited per transplantation site in small animal models is significantly smaller than in human patients, which in turn challenges the limits of the spatial resolution of any imaging technique. 

The aim of image acquisition in this proof-of-principle study, conducted in a small animal model, is to detect small clusters of transplanted cells at the transplantation target location and, potentially, track small clusters of transplanted cells which have migrated out of the target zone. Synchrotron imaging techniques, taking advantage of high photon flux and a highly collimated beam as well as of high-resolution detectors, can yield the high spatial resolution required to detect small clusters of transplanted cells and track their migration. We have shown previously that gold nanoparticles (GNPs) can be used to render implanted tumor cells’ X-ray opaque for synchrotron imaging [21,22]. We have now designed an experiment to explore the suitability of a synchrotron-based microCT technique to detect GNP-loaded transplants in a small animal model of neurotransplantation in HD. 

## 2. Materials and Methods

All animals were housed and cared for in a temperature-regulated animal facility exposed to a 12 h light/dark cycle. The experiments were performed in accordance with the guidelines of the Swiss and German Councils on Animal Care. The study was specifically approved by the Institutional Animal Care and Use Committees of Freiburg University Medical Center and by the authorities of the Land Baden-Württemberg (permit G-10/104, 2013).

### 2.1. Striatal Lesioning in the Rat Model

The lesioning and grafting procedures have been described previously [23]. Briefly, four adult female Sprague Dawley rats received a unilateral left-sided striatal lesion under general anesthesia, which was induced and upheld by Isoflurane (Abbott, 20097 Hamburg, Germany; 1–2% isoflurane in 2 L/min O_2_). The heads of the animals were fixed in a stereotactic frame (Kopf Instruments, Tujunga, CA, USA) with the nose bar set 2.3 mm below the interaural line. The heads were shaved and disinfected with Softasept, then a straight incision was made along the mid-sagittal line; the skin over the left side of the head was retracted and two burr holes were placed in the skull bone. The injection coordinates (in mm) with respect to the bregma were: AP_1_ = −0.4, ML_1_ = 3.7, DV_1_ = 5.2 and AP_2_ = 1.2, ML_2_ = 2.9, DV_2_ = 4.2, where AP indicates anterior–posterior; ML, medial–lateral; and DV, dorsal–ventral from the position below the dura. A total of 4 × 0.25 µL deposits of 0.12 M quinolinic acid (QA, Sigma, Ronkonkoma, NY, USA) dissolved in 0.1 M phosphate-buffered saline (PBS) at a pH of 7.4 were injected over a period of 1 min 40 s through a 30 G stainless steel cannula connected to a microdrive infusion pump. Two injections were applied through each of the two burr holes (i.e., 2 tracks were created, with 2 deposits of QA each). A 2 min interval was observed between the end of the injection procedure and removing the cannula from the injection position, to allow for diffusion of the fluid into the target tissue and to prevent backwash of the QA through the injection channels. The animals were then allowed to recover. At the QA injection sites, lesions developed over the duration of one week, after which the transplantation procedure with rat embryonic material was scheduled.

### 2.2. Dissection of Rat Embryonic Whole Ganglionic Eminences (wGEs)

As donor material for the neurotransplants, the whole ganglionic eminences (wGEs) of embryonic E13–14 Sprague Dawley rat embryos were used. One pregnant female Sprague Dawley rat, obtained from Charles River Germany, was overdosed with a Ketamine–Xylazine cocktail (Ketamine 150 mg/kg, Xylazine hydrochloride 15 mg/kg; Bayer, Germany). The abdomen was disinfected with 70% ethanol and opened by a V-shaped incision. The uterus was carefully dissected, washed twice in 1× phosphate-buffered saline (PBS, 10010-015, Gibco, Billings, MT, USA) and placed in a culture dish containing Dulbecco’s modified Eagle medium (DMEM/F12, 21331-025, Gibco). The individual embryos were delivered first from the uterus and secondly from the yolk sac, then placed into another culture dish with fresh DMEM. After the crown-rump length (CRL) of each embryo was measured to ascertain the developmental age, each embryonal head was opened under the dissection microscope from the posterior aspect along the midline, both wGEs were dissected carefully and placed in another culture dish with fresh DMEM. 

Once all wGEs were dissected, the tissue was aspirated and suspended in 10 mL of DMEM in a sterile 15 mL Falcon tube. After the tissue had settled on the bottom of the tube, approx. 9.5 mL of the supernatant DMEM was carefully aspirated. The tissue was then homogenized in the remaining DMEM using a 200 µL pipette tip. In our experience, a strict single-cell solution is not required for the success of cell transplantation in this model. The cell cluster suspension was finally topped up to a total of 10 mL with 0.05% DNASE-HBSS (DN-25, Sigma; CO 1341170-138, Gibco). Cells were counted using a hematocytometer (Neubauer chamber) to obtain an estimate of the material available for transplantation. Using trypan blue (T8154, Sigma), the overall vitality was determined to be over 95%. 

### 2.3. Tissue Culture and GNP Exposure

The aim of this step was to load the cells obtained from the embryonal wGEs with gold nanoparticles (GNPs), which would render the position of these cells radiopaque and thus detectable in X-ray imaging procedures.

Approximately 2.5 million cells suspended in 0.05% DNASE-HBSS were placed into a 95 mm diameter culture dish and 0.5 mL of GNP stock solution was added. Approximately 500,000 cells were seeded with the 0.05% DNASE-HBSS into a separate culture dish to use them for naïve transplants in the control animal. The cultures were placed into a humidified standard incubator (37 °C, 5% CO_2_).

The cells were harvested 22 h after seeding. By this time, the cells adhered to the bottom of the culture dish. The supernatant was gently aspirated, replaced by fresh HBSS and placed in the incubator for another 20 min. Following this, they were gently detached using a cell scraper and flushed off the bottom of the culture dish. The GNP-loaded cell cluster suspension was aspirated, placed into a sterile 15 mL Falcon tube and centrifuged at 1000 rpm for 4 min. The supernatant was carefully aspirated, the pellet was topped up with 10 mL of 0.05% DNASE-HBSS, gently broken up and resuspended. The cells were counted, following which they were again centrifuged and the supernatant was aspirated. These two centrifugation steps served the purpose of removing GNPs from the outer surfaces of the cells. Cell vitality was determined to be approximately 70%. The cells were then resuspended in DMEM for transplantation at a concentration of 100,000 cells/µL. For the naïve control, the same procedure was followed, save for the GNP loading.

Two samples of cells from the same stock, containing approx. 10^6^ cells each, were submitted to atomic absorption spectroscopy (ASS). The gold content was on average 3452 ppb/10^6^ cells (ca 42 µg/10^6^ cells).

### 2.4. Transplantation in the Rat Model

One week after creating the striatal lesions, three of the four lesioned animals received transplants of GNP-loaded cells. The remaining lesioned animal received a naïve cell transplant of equal size and thus served as control in the imaging procedure. 

**Figure 1:**
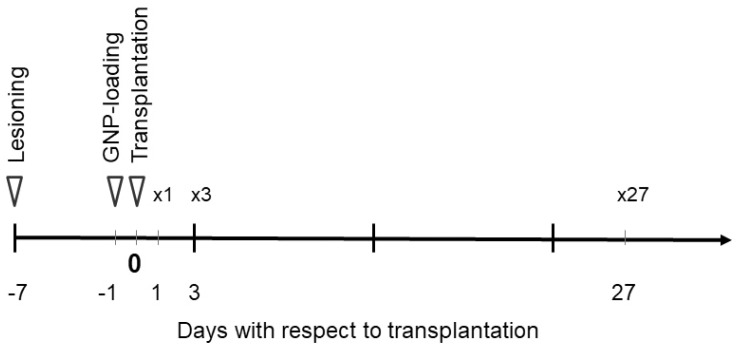
Timeline schematic of the small animal model used in this study, including the striatal lesioning, GNP loading, transplantation of embryonic material and the euthanasia in preparation of the imaging experiments on day 1 (×1), day 3 (×3) and day 27 (×27) after transplantation.

In all four animals, the transplants consisted of two deposits of 1.5 µL of a cell suspension containing 150,000 cells each (i.e., the total volume of the transplanted material was 300,000 cells in 3 µL per animal). Similar to the lesioning procedure, an interval between the end of the injection procedure and withdrawing the needle used for transplantation was observed to ensure the distribution of the transplanted cell suspension in the target area and avoid backwash. The transplantation coordinates with respect to the bregma were AP = 0.4 mm, ML = 3.3 mm, DV = 5.0 and 4.4 mm. The timeline of the individual steps required to create this model is shown in Figure 1.

The animals transplanted with GNP-loaded cells were euthanized at 24 h (1 day), 72 h (3 days) and 27 days after transplantation, respectively. The animal transplanted with naïve cells was sacrificed at 27 days after transplantation to ensure that we imaged the largest transplant, which would potentially increase the chance of visualizing it. The rats were terminally anesthetized and transcardially perfused with 300 mL of ice-cold 1× PBS, followed by 300 mL of ice-cold 4% paraformaldehyde (PFA). The head was then separated from the body and kept in the fridge until further use.

### 2.5. Synchrotron Radiation-Based CT Image Acquisition

Overview images and local tomography images were acquired at the TOMCAT beamline [24] of the Swiss Light Source (SLS), a third-generation synchrotron facility operating at an electron energy of 2.4 GeV and a ring current of 400 mA at the time of image acquisition. A double-bounce multilayer monochromator was used to select the desired energy from the emission spectrum of the 2.9 T bending magnet. As X-ray detector, a 20 µm thick LuAG scintillator was used, coupled via exchangeable objectives to a CCD camera (PCO.2000, PCO AG, Germany). The tomographic datasets were reconstructed using the TOMCAT processing pipeline with in-house software on a Linux cluster [25]. For three-dimensional image rendering, the open source viewer OSIRIX [26] and Drishti [27] were used.

The heads of the transplanted animals were individually mounted upright on a plastic plate in front of the detector. An energy of 25 keV was selected to account for the strong absorption of the bone. In a first step, the entire head was scanned with a magnification of 1.25×, yielding a pixel size of 5.92 µm and a field of view (FOW) of 12.1 mm. This overview scan was acquired with a total of 1000 angular projections (angular step size 0.18°) and with an exposure time of 250 ms/frame. In a second step, a local tomography scan was acquired, the center position was calculated from the position of the visible cells in the overview scan. The magnification in this local tomography scan was 20× at the position of the transplant, with a total of 2000 tomographic projections and a duration of 120 ms/frame (pixel size 0.37 µm, FOV 750 µm). The overall scan time was between 4 and 5 min for both the overview and the high-resolution scans.

### 2.6. MR Image Acquisition

Different from CT images, soft tissue structures can be visualized in great detail in MRI images even without specific contrast enhancement. Data were acquired on a small animal 9.4 Tesla BioSpec MRI scanner system with BGA12S gradients (Bruker BioSpin GmbH, Karlsruhe, Germany). The sequence used was a T1-weighted 3D FLASH with TE = 10 ms, TR = 34 ms and the flip angle α = 30 degrees. The voxel was an isotropic cube of 60 μm side and the field of view was 15.3 × 11.9 × 15.3 mm^3^. The images were converted to DICOM format and reconstructed using IMPAX EE software VIII (AGFA Healthcare, Mortsel, Belgium). Image acquisition time was approximately 14 h per animal.

### 2.7. Immunohistochemistry

For technical reasons, the immunochemistry images shown in this study are not from the 4 animals used in the imaging study but taken from two animals lesioned in an identical protocol. Only one of these animals was also transplanted with embryonic material, with naïve cells (without GNP loading). The rats were terminally anesthetized and transcardially perfused with 300 mL of ice-cold 1× PBS, followed by 300 mL ice-cold 4% PFA. The brains were carefully removed from the skull and post-fixed by immersion in 4% PFA for 12 h. They were then transferred into a 30% sucrose solution and left there at 4 °C until they had sunk to the bottom of the dish. Coronal sections of 40 μm thickness were cut on a cryomicrotome and collected in PBS in a multiwell-plate. 

NeuN-immunostaining was conducted to visualize mature neurons. To achieve this, endogenous peroxidase activity was quenched by incubating the sections for 5 min in 3% H_2_O_2_ and 10% methanol in PBS. Nonspecific binding was blocked by a 1 h pre-incubation in 5% normal serum containing 0.25% Triton X-100 in PBS. The tissue sections were incubated with the primary antibody (mouse anti-NeuN, 1:250; Merck Millipore MAB377, Darmstadt, Germany) overnight at room temperature. Then, the sections were rinsed 3 times with 1× PBS, followed by a 1 h incubation in the appropriate biotinylated secondary antibody (rabbit anti-mouse, IgG biotinylated, 1:200, Dako E0464, Glostrup, Denmark) and 1 h incubation with avidin–biotin peroxidase solution (ABC Elite; Vector Laboratories). Finally, 3,3-diaminobenzidine (DAB; Merck, Darmstadt, Germany) and 0.01% H_2_O_2_ were used to develop the color reaction. Sections were mounted on slides, air-dried and cover slipped.

## 3. Results

### 3.1. Comparison of Imaging Modalities

Transplants were detected in both the overview and local tomography images in all animals that received GNP-loaded transplants. No transplants could be detected with either method in the animal that had received naïve cell transplants, i.e., transplants without GNPs as means of contrast enhancement. This was not unexpected since there is no density difference between the transplant and the normal brain tissue. Therefore, no contrast between the two soft tissue components is expected. 

**Figure 2:**
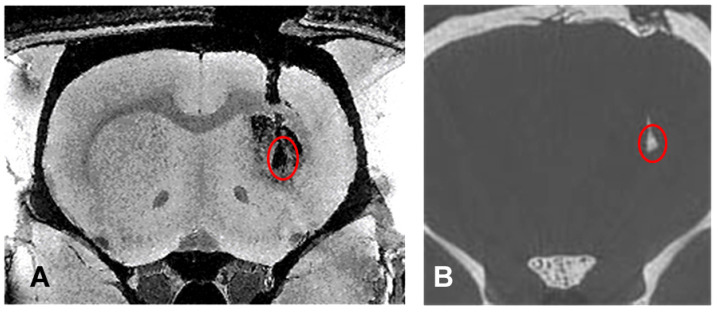
MRI (**A**) and synchrotron X-ray microCT overview images (**B**), obtained from the same animal, transplanted with GNP-loaded cells and sacrificed at 27 days after transplantation. The lesion (within the red circle) is shown in both images in equal size and shape. However, the information detail offered by both images is different. While the MR image shows the extent of the lesion, position, size and shape of the transplant as well as the needle tract left after transplantation, the microCT image shows position, size and shape of the transplant as well as the fact that some cells have migrated and proliferated into the needle track. The latter information cannot be deduced from the MRI.

Both the MR and the microCT overview images inform about position, shape and size of the transplants in a comparable manner (Figure 2). However, there is additional information which is specific to both types of image modality. 

The MRI shows in detailed structure the two lesions created by the QA injections and the structural defect created by the transplantation needle track (Figure 2A). The transplant can be seen as a dark void caused by the GNP-loaded cells between the two lesions created by QA. In the synchrotron microCT, neither detailed brain structures nor the lesions can be seen. However, additional information, the deposition of contrast-enhancing material is noticed in the caudal part of the needle track, suggesting the presence of the transplanted material, either as misplaced material at the time of transplantation or due to the migration and proliferation of the originally transplanted material into this location (Figure 2B). This is important information which cannot be obtained from the MRI.

**Figure 3:**
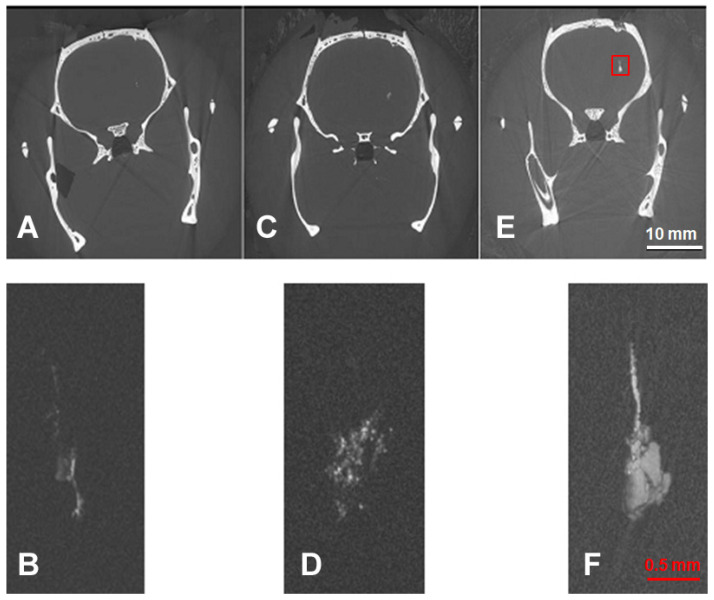
Synchrotron CT: overview (**A**,**C**,**E**) and local tomography images (**B**,**D**,**F**). Images obtained of animals sacrificed at 1 day (**A**,**B**), 3 days (**C**,**D**) and 27 days (**E**,**F**) after transplantation. Lesion outlined in the red box in (**E**).

No small cell clusters can be identified in either the MR image nor the synchrotron microCT overview image. Only the extremely high spatial resolution observed in the local tomography images obtained at the synchrotron allowed the detection of small cell clusters. The volume increase in the transplants can be followed for almost one month, between day 1 and day 27 after transplantation (Figure 3). The total amount of GNPs is the same in all three transplants. Thus, the visible volume increase in the transplants suggests that, during the proliferation process of the transplants, the GNPs are distributed between the daughter cells of the originally transplanted material and that the GNP content is still sufficient for detection in the TOMCAT microCT system after several proliferation cycles. The lesions cannot be identified in either the overview or the local tomography images. As already seen in the overview image at day 27 after transplantation, GNP-enhanced material is seen along the transplantation track. Beyond this general statement, the high spatial resolution of the microCT local tomography shows that the enhancement decreases with increasing distance from the transplantation site. This suggests that, rather than representing misplaced material from the transplantation, some of the GNP-loaded cells have migrated and proliferated along the injection track and thus render it partially visible in the X-rays of the microCT.

### 3.2. Histology

**Figure 4:**
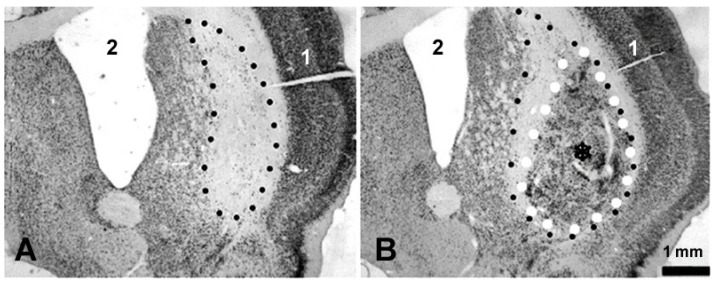
Photomicrograph depicting the effects of striatal injections of quinolinic acid (**A**) and the implantation of the embryonic striatal tissue into a previously lesioned striatum (**B**). A needle track from the lesioning procedure can be seen (1). The sections are stained with the neuron-specific marker, NeuN. Enlarged ventricles (2) and neuronal loss, mirrored in a reduction in staining in the striatum, are induced by the lesion (delineated with black dots (**A**,**B**)). The grafted tissue, identified by the asterisk and delineated with white dots, is rich with neurons (**B**).

The animals for the imaging study and the animals for the immunochemistry were lesioned and transplanted according to the same protocol but not on the same day. Nevertheless, both the tear-drop shape and the size of the transplant are very similar to those seen in the microCT and MRI images (Figure 4B) obtained from the animals prepared for the synchrotron imaging study. This attests to the reproducibility of the procedure.

## 4. Discussion

Preclinical cell transplantation studies with promising results have been reported for small animal models of heart failure [28] and intraventricular hemorrhage [29], multiple sclerosis, spinal cord injury and stroke [30,31]. Already in 2000, Björklund hypothesized that embryonic stem cells and neural stem cells might provide renewable sources of cells for therapeutic purposes as a powerful alternative to primary fetal CNS tissue in clinical transplantation protocols [32]. 

With non-malignant cells transplanted, one would expect a self-limitation of proliferation. However, the slow-down of the proliferation rate can be easily misjudged and, therefore, tumor growth is a potential risk associated with such a procedure [33]. This can occur at the target zone or, if transplanted cells migrate out of the target zone, as metastatic growth in unexpected locations. The case of a patient developing a symptomatic intraspinal, graft-derived mass eight years after receiving a transplant of olfactory ensheathing cells within a clinical study protocol to treat spinal cord trauma has been reported [34]. Another case of a patient with overgrowth (graft-derived tumor formation) has been described after transplantation for Huntington’s disease [11]. A method for cell tracking to allow for the early detection and, if necessary, intervention before a patient becomes symptomatic from a graft-derived tumor formation would be extremely suitable both in research models and, potentially, also in patients.

The intriguing new aspect of synchrotron microCT is that the local tomography mode provides us with an option to detect small cell clusters within the transplant, as opposed to the seemingly homogeneous transplant seen in MRI images. Based on the sharply contrasted delineation of the transplant from surrounding tissue and the extremely high spatial resolution, potential future applications of this synchrotron-based imaging method would be long-term studies in small animal models of cell transplantation, assessing both local transplant development and cell migration out of the target region. Both aspects are of importance in transplantation surgery. 

Superparamagnetic iron oxides (SPIOs) have been tested recently as an intracellular marker for transplanted MSC in a canine model [35]. While the SPIO-labelled grafts were detectable in both MRI and histology, the authors themselves question whether the method could be sensitive enough to detect small cell clusters or even single cells. To explore which type of research question can be addressed best using GNP-loaded cells and microCT, or where SPIO and susceptibility-weighted MRI might provide the better solution, further studies should be conducted. The combination of synchrotron CT and GNPs as an intracellular marker would allow for the long-term detection of small groups of transplanted cells. We have shown previously that the resolution of small cell clusters is possible and used GNPs in vivo as intracellular markers in a small animal model of malignant brain tumor [22]. The inherent challenge in those experiments was the high mitotic activity of the implanted tumor cells, which can cause the cellular GNP content to fall below the detectable threshold after several mitotic cycles. The harvested E13/E14 whole ganglionic eminence cells used in our current model are all progenitor cells committed to become mainly striatal, but also to non-striatal neurons. They are nearly post-mitotic and in most cases, very little division occurs following transplantation [36]. Thus, the amount of GNPs per cell is more likely to remain above the detection limit. This assumption is supported by the fact that, in our current study, even at 27 days after transplantation, the CT images of the brain with GNP-enhanced transplants show a high correlation in size and shape with the transplants seen in the small animal MRI. Therefore, one can assume that most of the transplanted cells still contain a sufficient number of GNPs to render them above the detection level, although the transplant has increased in size significantly within the 27 days after transplantation. Cell tracking by microCT in vivo has been attempted. Gold nanocomplexes were used to label mesenchymal stem cells [37]. The observation time, however, was limited to 5 days and the detection limit was higher than 1,000 cells. The contrast was already significantly decreasing during this relatively short observation period. In another interesting study, considered long-term tracking by its authors, GNP-labelled human stem cells were followed for 10 days after transplantation to study the fate of the transplanted stem cells in a small animal model of pulmonary fibrosis [38]. No estimate of the X-ray dose administered in either of these studies was provided. In either case, the observation time was significantly shorter than in our current study.

This pilot study was designed to address the question whether the synchrotron microCT was sensitive enough as a method to follow the size increase in the transplants over time. Despite the small number of animals used, we were able to show that a continuous size increase in the transplants from GNP-loaded striatal progenitor cells obtained from wGEs of E13–14 embryos can be detected between 1 and 27 days after transplantation in a small animal model of Huntington’s disease in the synchrotron microCT, whereas transplants generated from naïve cells cannot be distinguished. While the CT scanner used in everyday diagnostic procedures in human patients offers standardized equipment components and routine scripts for image acquisition, this is not the case with synchrotron-based microCT. At the synchrotron, the equipment can be set up in different constellations to suit a specific research question. If the use of GNP-loaded cells for tracking is used more frequently in the future, a permanent setup with fixed equipment positions might be developed to save setup time, and user-friendly software macros would be written.

Our imaging method is suitable for an extremely detailed 3D rendering of the transplant structures in small animal models ex vivo/post mortem. However, the X-ray doses required to achieve the high spatial resolution for small cell clusters is considerably high. An estimate for the dose deposition rate at 25 keV in brain tissue at the TOMCAT beamline was quoted by the authors of [39] to be approximately 6.9 Gy/s; for an overview scan, the head was exposed 250 s to the beam, and thus an approximate dose of 1.7 kGy was delivered to the brain. This is clearly beyond the limit for a repeated follow-up imaging in vivo. As a method for a non-destructive imaging of whole brains ex vivo, however, this imaging protocol can be extremely useful. Future therapeutic visions might focus on transplants generated from mesenchymal stem cells and pluripotent stem cells to induce regenerative effects, to avoid logistic problems and ethics discussions inherent in the use of fetal tissue grafts [40]. Our protocol could be modified for use with those cell entities, rendering the combined approach of non-destructive three-dimensional imaging for morphological analysis at high spatial resolution and immunohistochemistry—to assess the function and integration of the transplants into the host environment—a very elegant analytic combination in the experimental workup.

## 5. Conclusions

Synchrotron-based microCT is an intriguing method for the non-destructive three-dimensional analysis of small cell clusters in neurotransplants in a small animal model of Huntington’s disease. Owing to the high X-ray doses deposited in the target tissue during the process of image acquisition, however, at the current stage, the technique is suited exclusively for ex vivo/post mortem analysis. 

## Data Availability

Original data are available on demand from the corresponding and senior authors.
